# McGugin on Erysipelas

**Published:** 1853-08

**Authors:** D. L. M’Gugin

**Affiliations:** Professor of Physiology, Pathology and Clinical Medicine in the Medical Department of the Iowa University


### *A Case of Phlegmonous and Gangrenous Erysipelas.*

BY D. L. M'GUGIN, M. D.,

Professor of Physiology, Pathology and Clinical Medicine in the Medical Department of the Iowa University.

Mr. Morton Grocer of this city, aged 43, and of spare habit was attacked on the 11th of April last with rigors followed by fever, pain in the head, hot skin and an insatiable thirst. There was at the same time a spot observable upon the arm about the middle of and over the biceps muscle highly inflamed attended with a burning pain. He was restless, the mouth and fauces dry and secreting a tough viscid mucus difficult to be removed by the ordinary efforts.

The fever increased, thirst continued urgent, the skin attained a higher temperature and the inflamed part upon the arm extended its limits and continued to spread more rapidly to the extremity than to the shoulder, the swellıng as rapidly increased and with the augmentation of these symptoms the suffering was also much enhanced.

This continued until the 16th when I first saw him. At this time the face was expressive of anxiety, a livid circle below the eye, the epithelial covering of the lips was also livid and encrusted, there were sordes on the teeth, the tongue and fauces presented a leaden lividity the former covered with a fur almost black and the latter inflamed, the lower extremities cold, the skin dry and rather cold for want of capillary circulation, bowels costive, tenderness in the epigastrium and throughout the track of the color which was distended, with gaseous and fœcal matter, the pulse small, contracted and from 115 to 120 in frequency. The urine was dark brown and fœted and voided with pain and diffiulty.

The condition of the arm next claimed attention. This was livid and swollen to the extremities of the fingers measuring above the elbow nearly 14 inches in circumference. On the inner side of the arm there were several bullæ or blebs which became confluent and burst, discharging their contents leaving a death-colored surface denuded of its cuticular covering and in the spot where the inflammation first appeared there were two or more phlyctæna filled with sanguineous serum. There were also several echymotic spots over the hand and the under surface of the arm. Whilst the skin kept its integrity it retained the great effusion of serum beneath it but now there were numerous breaks or fissures through which that fluid slowly escaped.

It was very evident that not only the skin, and the cellular tissue beneath, had been involved in the most destructive grade of inflammation, but it had also reached down to the sub-aponeurotic cellular tissue upon which it was then exerting its power. There was watchfulness, jactitation, and during the night there was delirium.

*Prognosis.*—Simple erysipelas is usually easily managed, but in this case the symptoms augured unfavorably. The great loss of the dynamic forces and the disorganization and destruction of so much structure to be restored by the feeble vital powers still remaining, were circumstances truly discouraging. How far these enfeebled powers would go toward restoration, and how long they might be stimulated to continue their efforts, a short time would determine.—An intelligent view of the peculiar nature or the pathology of the disease as indicated by the phenomena, and the results in a majority of such cases, left but little to hope for in this. Already the system was sinking under the influence of the morbific poison which was rapidly augmenting by absorption from the mass of disorganized tissues clinging to it, and by its presence involving others in its devastating progress, presenting a proof that the recuperative powers, instead of asserting their prerogative or effecting any thing favorable, were yielding submission to the power of the disease.

*Treatment.*—The repeated exhibition of ol. ricini. with ol. terebinth. and an occasional enema of the same with thin strach water, overcame the constipated state of the bowels and brought away dark and fœtid discharges. As soon as this was accomplished, the terebinthinate emulsion was given every 4 hours, intermediate to which a pill composed as follows:

℞ Ext. cinchona precip. half drachm,
Gum opii. pulv. three grains,
Pulv. camph. one scruple,
Mix and make ten pills,

Of these one every 3 hours. About half an inch of the inflamed skin with an equal width of the sound, was painted with the saturated tincture of iodine and the entire limb enveloped in a linen cloth saturated with a solution of *sulph. ferri.* in the proportion of ℥j. to the pint of water and kept constantly moist with it.

At the end of 24 hours, although the diseased tissues exhibited greater evidences of disorganization, there was no greater frequency of the pulse, the lips and edges of the tongue were less intensely red, the dark fur was inclined to dissipate, and the patient rested better.

There was less starting, less anxiety and jactitation, and it was evident from all the symptoms that the dynamic forces were awakened to a renewed effort and sustaining themselves better; yet the morbific poison was still circulating and supplied in quantities too great for the depurators to throw off. The fetor of the diseased mass was yet too considerable, although the before mentioned solution had been faithfully persevered in. The pills were continued but the emulsion was laid aside for the following by Watson:

℞ Chlorate Potas. ʒi,

Hydrochloric acid ʒi,

Water ℥ij,

Add the acid to the water, then the potass. To a pint of water add ʒij of this mixture of which a table spoonful was given every two and four hours according to the indications.

The chlorinated soda in solution combined with creosote was used externally to the arm, now exhibiting a mass of disorganization, which lessened the fetor. Removing such as became loose and detached from time to time, the chlor. soda and creosote were continued, so also were the other remedies and in addition to the foregoing, the infusion of cinchona, as well as the frequent use of the chlorine mix. as a gargle to the throat which still continued to be inflamed. As the inflammation had dipped down beneath the facia and involved the inter-muscular tissue, a diffused abcess formed and as soon as fluctuation was evident a bistoury passed through the facia gave discharge to a quantity of sero-prulent matter at different times. The sloughing continued in some spots very deeply, the orifice continued to discharge, the pulse continued to grow slower and fuller, the tongue in a few weeks cleaned off, granulations began to show themselves in time, the hemorrhage from the bowels accompanied with great fetor, which took place in consequence of disorganization of their mucous lining was arrested by enematis of decoct. of oak bark with acetata plumb and did not recur, the tenderness of the epigastrium, over the track of the colon and over the hypogaster was relieved and the appetite began to crave food. Œdema and the exuberant granulations of the sloughs were treated by compression, in the course of time the use of *brown stout* was substituted for the chlorine mix. and the other remedies were gradually withdrawn from time to time. The use of the iodine arrested the onward march of the inflammation in the skin, the turpentine and castor oil was used to regulate the when necessary, which were exhibited in equal quantities, the extremities were kept warm by sand heated, put into bags and kept to the feet. We may profitably indulge in some speculations into the pathological condition of the general system and the morbid anatomy of the parts now particularly affected. That the mind may be led to institute enquiries and indulge in reflections constitute one of the objects of reporting the particulars in each case.

In simple erysipelas or erythema, the skin alone is inflamed. This form does not require much attention, unless it suddenly leaves the surface and attacks a vital organ. Even then, in a majority of cases, it may be restored by the application of stimulants or warmth. A more aggravated case is where the inflammation attacks the areolar tissue beneath, but exterior to the fascia. In these tissues we have often abscesses formed. Again, in other cases, we may have the phlegmonous and the gangrenous varieties, existing independent of each other, and in other cases they are combined. Where the strength of the subject is well maintained under the phlegmonous form and the parts do not sink down into a gangrenous condition from the force of the invasion, then the inflammation may dip down from the skin to the celular tissue, to the intermuscular tissue, thence to the periostium, and finally to the bone itself, which it may destroy. In a majority of cases, however, they would prove gangrenous from the force of the onslaught, the system then would succumb, and the patient ultimately, nay, sometimes very speedily, sink. The inflammation in these cases is peculiar, but in what does this peculiarity consist? Is it that it depends upon phlebitis? It is well known to those who have prosecuted enquiries into the morbid anatomy of this peculiar inflammation, that the inner coats of the veins exhibit marks of a high grade of inflammation. The fact of its appearance most frequently in parts of the greatest vascularity and that other well known fact that pus has been found deposited in certain organs, as the liver, the lungs, and elsewhere, having been carried there by the circulation, would seem to favor this conclusion. Valpeau maintained it was phlebitis, and Rayer, with others, have remarked the peculiar appearance which the inner coats of the veins wore in erysipelas. Others point to the fact as proof of this position, that the most violent forms of it have been produced by the slightest scratch on the finger. The similarity of the morbid anatomy in this and in uterine phlebitis, in which pus is carried to various tissues and there deposited, is another fact worthy of considerate attention. M. Blandin, of Paris, regards it as being confined to the lymphatics in the celular tissue. Although the lymphatics are often involved in common with other tissues, it is however questionable whether they are primarily diseased.

In these cases where the inflammation passes readily into the asthenic state, and attended with a great loss of the vital powers, the pus is seldom laudable, there is no coagulable lymph, but the matter formed is sero-purulent, and is extensively diffused. In this case we have related there were discharged a number of shreds, indicating the destruction of the intermuscular tissue. And here I would remark, that because of the destruction of this tissue with the facia of the several muscles, adhesions, between the fibres of the muscles, would doubtless have resulted, with consequent immobility of the limb. To avoid this, he was instructed to flex and extend his arm as often as prudent, in order to prevent the formation of adhering bands, which happily accomplished the object.

Early in this case much good would have resulted from incisions or punctures, but at the time we saw him they would have availed nothing, because of the numerous fissures which favored the escape of serum. Speedily following this the entire cuticle sloughed off, leaving in many places deep sloughs into the celullar tissue. A difference of opinion and practice obtains among different leading men, with regard to the propriety of extensive and free incisions.

Mr. Copeland, Hutchison and others advocate the free incisions. If it were confined to the lower limb, and extending upward even to the crest of the ilium, then he would incise from the trochanter to the toe. Owing to the fullness of the cellular tissue by the effusion an extensive and frightful wound would appear. This could not long continue for the fullness of the tissues acting as a cause of distension, the serum being discharged, the limb would lessen in diameter, and so also would the wound diminish in size.

The propriety of these free incisions is regarded by many as very doubtful. If resorted to, it must be in severe, and recent cases, when the condition of the system would bear and the extent and severity of the inflammation demand the antiphlogistic treatment. But if the constitutional disturbance be great and the tendency be to a depression of the general system, they are then to be avoided and the small incisions or punctures to be preferred. There are several peculiarities in this form of inflammation, and one of these is the tendency rapidly to become asthenic, followed soon by an extensive destruction of the tissues. This tendency modified and influenced by some constitutional disturbance as in traumatic erysipelas, or by a present epidemic influence, exhorts to great care and caution lest a sudden loss of the vital forces would obtain from bold measures. Hemorrhage often occurs, and if it do not, we may inflict a fatal shock upon the nervous system. Sloughing, extensive and devastating has occurred more extensively perhaps than if the incisions had been less free.

In this case there was a great depression in the system by the presence of the morbific poison circulating in the blood and inflicting its mischief upon the great nervous centre, the brain No wonder then we found a great loss of the biotic forces. The system was sustained by the tonics and stimulants while the active poison was neutralized by the chlorines used externally and internally. A decidedly favorable effect was observable from their use and were steadily persisted in, and so marked was the result that I regarded their use as the most potent influence exerted in the case, and that the favorable result was attributable more to their use than to any thing else, and perhaps to all else combined.

